# Determination of the Immunomodulatory Role of OTOP2 in Colon Adenocarcinoma

**DOI:** 10.7150/jca.95622

**Published:** 2024-07-16

**Authors:** Chenglu Lu, Shuai Chen, Shasha Liu, huimin Liu, Lin Sun, Yan Sun

**Affiliations:** 1 Department of Pathology, National Clinical Research Centre for Cancer, Key Laboratory of Cancer Prevention and Therapy, Tianjin's Clinical Research Centre for Cancer, Tianjin Medical University Cancer Institute and Hospital, Huanhu West Road, Hexi District, Tianjin 300060, China.; 2 Department of Pathology, Affiliated Hospital of Jining Medical University, Jining 272100, Shandong, China.

**Keywords:** OTOP2, Colon adenocarcinoma, Tumor suppressor, Immunomodulatory, Prognosis.

## Abstract

**Background:** Otopetrin 2 (OTOP2) is a conserved ion channel protein that regulates cell signaling, growth, and development. Although the role of OTOP2 in tumor suppression has been reported in several studies of colon adenocarcinoma (COAD), characterized its immunomodulatory effects on tumors.

**Methods:** We conducted a thorough analysis of OTOP2 expression and its association with clinicopathological characteristics, immune-related pathways, and immune-related molecules in individuals with COAD using data from The Cancer Genome Atlas (TCGA) and confirmed the findings with tissue microarrays (TMAs). We conducted *in vitro* assays to demonstrate the tumor suppressive effect of OTOP2 in COAD cells.

**Results:** OTOP2 expression was abnormal in multiple types of tumors and was significantly downregulated in patients with COAD (*P*<0.001). Moreover, the presence of OTOP2 was linked to enhanced survival in individuals diagnosed with COAD. *In vitro* experiments showed that OTOP2 suppressed cell proliferation, migration, invasion, and adhesion. Gene set enrichment analysis of the TCGA database indicated that OTOP2 was positively correlated with antigen presentation pathways and T cell responses. The immunophenoscore (IPS) indicated a positive correlation between OTOP2 expression and MHC molecule expression (*P*<0.001) as well as between OTOP2 expression and the number of effector cells (*P*<0.01). Immunohistochemical analysis of the TMAs revealed strong associations between OTOP2 expression and MHC-I, TAP1, and TAP2 expression, and between OTOP2 expression and CD8^+^ T cell infiltration in COAD patients.

**Conclusion:** In summary, our research emphasizes the role of OTOP2 as a tumor suppressor, suggesting its use as a prognostic indicator and predictor of response to immunotherapy in COAD patients.

## 1. Introduction

Colon adenocarcinoma (COAD) is the most common malignant tumor in the gastrointestinal tract. Since this disease is difficult to detect in the early stages, it often progresses to advanced stages before diagnosis^1^. Surgical treatment is the major treatment for COAD, but the clinical prognosis for stage III/IV patients has been found to be unfavorable, even with the addition of adjuvant therapies^2^. Therefore, identifying effective biomarkers is necessary to help doctors choose suitable treatments for COAD patients. Tumor tissues consist not only of cancer cells but also of a tumor microenvironment (TME) composed of endothelial, stromal, and immune cells, as well as cytokines and chemokines. The interactions between the TME and cancer cells can promote or hinder tumorigenesis, growth, metastasis, and response to therapy^3^. Among TME-infiltrated immune cells, T cell play a crucial role in antitumor immunity^4^. As a result, the identification of immune cell type, distribution, and abundance in relation to tumors is now widely acknowledged as important for predicting patient outcomes and response to treatment. Notably, CD8^+^ T cell based immune infiltration has proven to be a superior prognostic indicator for COAD^5^.

Recently, a novel superfamily of proton-selective ion channels, known as Otopetrins (OTOPs), has been identified^6^-^8^. Among the OTOPs, there are three members: OTOP1, OTOP2, and OTOP3. Among them, OTOP2 is conserved among species, is expressed in various tissues, and plays an important role in diseases of the gustatory and digestive systems; for example, high OTOP2 mutation can induce enteritis and COAD^9^, ^10^-^13^. Some research has suggested that OTOP2 could function as a gene that inhibits tumor growth in COAD. However, the connection between OTOP2 expression in COAD and the TME remains unknown. Thus, a comprehensive analysis of OTOP2 expression and its clinical importance, along with its correlation with CD8^+^ T cell, is needed.

This research involved a thorough examination of OTOP2 expression by utilizing The Cancer Genome Atlas (TCGA) database, revealing a decrease in gene expression in COAD and a connection between abnormal expression, OTOP2 mutations, and DNA methylation. In COAD,* in vitro* studies showed that OTOP2 suppressed the proliferation, migration, invasion, and adhesion of tumor cells. Notably, OTOP2 exhibited a positive correlation with pathways related to antigen presentation pathways and T cell responses. The immunophenoscore (IPS) indicated that the expression of OTOP2 was also positively associated with major histocompatibility complex (MHC) molecules and effector cells. These results were validated through immunohistochemical (IHC) staining of COAD on tissue microarrays (TMAs). Survival analysis in the TCGA, GEO and TMUCIH-COAD cohorts indicated that high OTOP2 expression was associated with increased overall survival (OS) in patients with COAD. This research emphasizes the significance of OTOP2 as a suppressor and a possible indicator of prognosis in COAD patients. This study highlighted the close connection between OTOP2 and components of the tumor immune microenvironment, suggesting that patients with high OTOP2 expression in COAD could benefit from immunotherapy.

## 2. Materials and methods

### 2.1. RNA-seq data and availability

**Figure 1:**
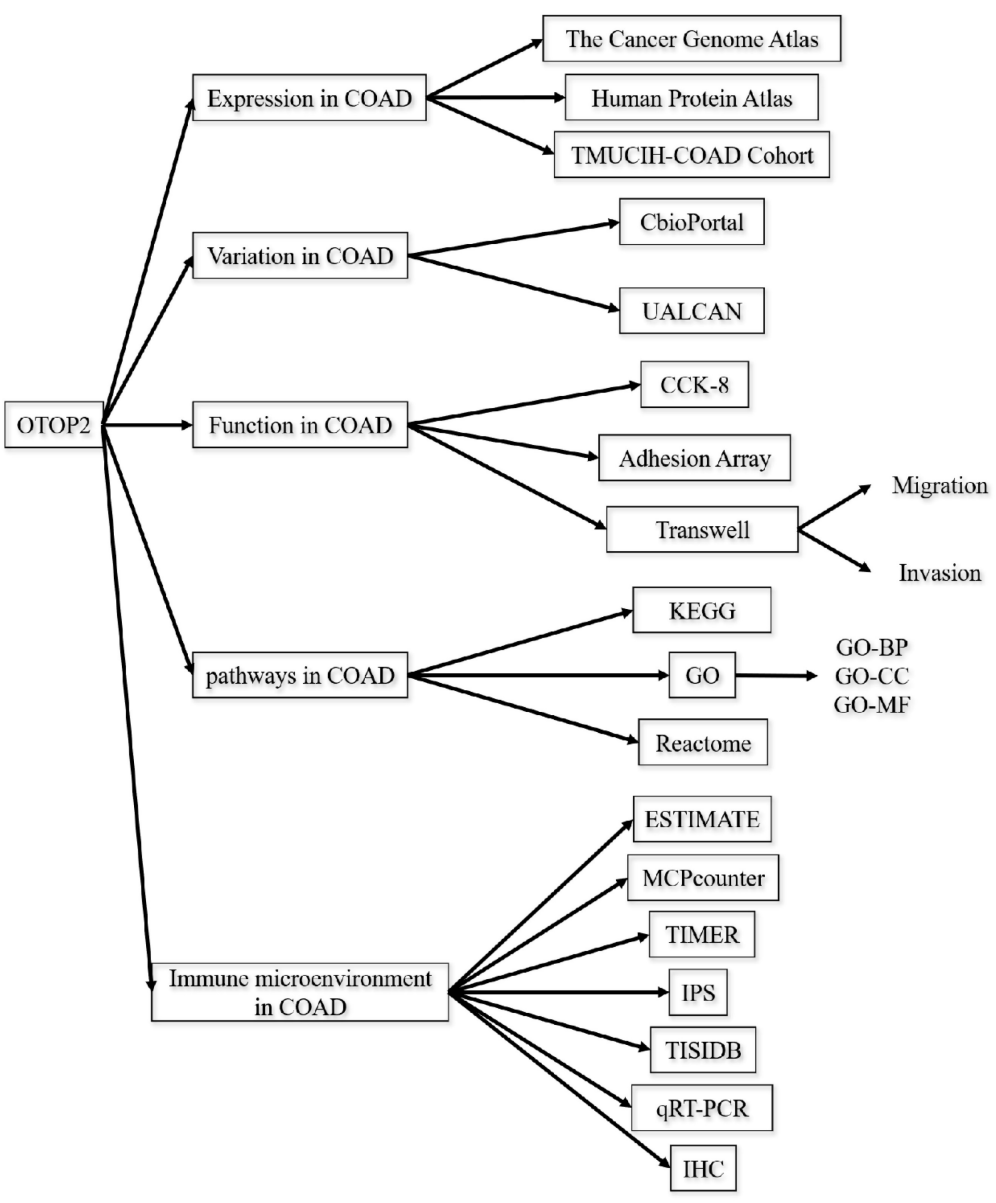
Schematic design of the study.

RNA-seq data were obtained from the TCGA (https://www.cancer.gov/ccg/research/genome-sequencing/tcga) and Gene Expression Omnibus (https://www.ncbi.nlm.nih.gov/geo/) databases. The original data were integrated using R software version 4.2.1, and the subsequent analysis was conducted utilizing the online web tools outlined in the following section. The flowchart of this study is displayed in Figure 1.

### 2.2. Analysis of OTOP2 mRNA expression in tumor and normal tissues

The OTOP2 mRNA expression data were explored via the SangerBox website (https://vip.sangerbox.com/)^14^,^15^. If the data in each group were normally distributed, two-sample Student's t tests were used; otherwise, the Mann-Whitney U test was used. The results were visualized and verified by R 4.2.1 and ggplot2.

### 2.3. Survival analysis

The prognostic significance of OTOP2 mRNA expression in the TCGA-COAD and GSE39582 cohorts was assessed using the PanCanSurvPlot database (https://smuonco.shinyapps.io/PanCanSurvPlot/)^16^. The clinical data of the TCGA-COAD are shown in Supplementary Table 1. According to PanCanSurvPlot, the cutoff value for classification into groups was the median mRNA expression of OTOP2.

### 2.4. Examination of gene location, copy number alternations and DNA methylation

The copy number alteration (CNA) features of OTOP2 were identified using cBioPortal (https://www.cbioportal.org/)^17^. Data on changes in frequency and CNA for TCGA-COAD were acquired through the modules “Cancer Types Summary” and “download”. The UALCAN platform (https://ualcan.path.uab.edu/index.html) was utilized to assess the extent of OTOP2 promoter methylation in both normal and primary tumor tissues^18^.

### 2.5. Analysis of the enrichment of functions

Differentially expressed genes (DEGs) were determined between the low and high OTOP2 mRNA expression groups using the 'limma' R package, with the conditions of |log2FC|>2.0 and P value<0.05. Enrichment analyses for Gene Ontology (GO), Kyoto Encyclopedia of Genes and Genomes (KEGG), and Reactome were conducted using the R package 'clusterProfiler'^19^-^21^. Functional pathways were visualized using gene set enrichment analysis (GSEA) with the reference file 'c2.cp.kegg v7.4.symbols.gmt' (https://www.gsea-msigdb.org/gsea/index.jsp). Statistical significance was determined by a false discovery rate (FDR) of less than 0.05.

### 2.6. Examination of immune cell infiltration

The OTOP2 mRNA was analyzed using single-sample GSEA (ssGSEA), MCPcounter, and IPS through the webtool for Comprehensive Analysis on Multi-Omics of Immunotherapy in Pancancer (CAMOIP, http://www.camoip.net), and the results were obtained from^22^-^24^. We downloaded correlation data from CAMOIP to specifically examine the relationship between the mRNA expression of OTOP2 and immune-related genes, including those involved in the expression of immunostimulants, immunosuppressors, and MHC molecules. To visualize our results, we utilized the R package ggplot2. Additionally, we utilized the CAMOIP database to determine the correlation between OTOP2 mRNA expression and the immune cell infiltration score in COAD patients in the TCGA-COAD cohort. We also collected data on the expression of eight common immune processing- and presentation-associated genes and explored their correlation with OTOP2 mRNA expression^25^. To analyze our findings, we used R software for statistical analysis and verified the results. Finally, we retrieved RNA-seq data from the TCGA-COAD cohort. The ESTIMATE, immune, and stromal scores were calculated from these data using the estimate R package^26^. TISIDB, a webtool that integrates numerous cancer datasets sourced from the TCGA database (http://cis.hku.hk/TISIDB/)^27^, was utilized to investigate the relationship between OTOP2 and molecules involved in antigen presenting.

### 2.7. Samples collected from the Cancer Institute & Hospital at Tianjin Medical University

The TMUCIH-COAD cohort consisted of 348 COAD tissues and their corresponding normal tissues obtained from the Department of Pathology at Tianjin Medical University Cancer Institute & Hospital between 2011 and 2015. We received institutional review board approval from Tianjin Medical University Cancer Institute and Hospital (Tianjin, China). All of the samples were obtained from inpatients who underwent surgery between January 2011 and December 2015, and none of the patients had received any chemotherapy or radiotherapy before the operation. We excluded patients with other malignancies, chronic diseases, or no available slides or blocks in the archives. Each participating study patient provided informed consent. The clinicopathological information of the patients was also collected (Supplementary Table 2). The study received approval from the Hospital Institutional Review Board at Tianjin Medical University.

### 2.8. TMAs and immunohistochemistry

TMAs were constructed as previously described^28^ and subjected to IHC staining and scoring. With the EnVision two-step procedure, IHC analysis was carried out on paraffin-embedded Transprimer antibodies from various sources, including anti-MHC class I (ab134189; Abcam, USA), anti-CD8 (EP334; Roche Diagnostics, USA), anti-TAP1 (PA5-80094; Thermo Fisher, USA), anti-TAP2 (PA5-37414), and anti-OTOP2 (bs-17535R; Bioss, China) antibodies. The process was executed following the guidelines provided by the reagents, including the necessary positive and negative controls. Positive expression of OTOP2 was identified by yellow or brown staining in the cytoplasm of tumor cells. MHC-I was mainly observed in the membrane, and TAP1 and TAP2 were found in both the cytoplasm and the membrane. The IHC score was evaluated by two independent pathologists. The staining intensity of the above markers was rated on a scale ranging from 0 to 3, with 0 indicating no staining, 1 indicating mild yellow, 2 indicating yellow, and 3 indicating brown. The percentage of positive cells was assessed on a 0 to 4 scale, with 0 indicating no positive cells, 1 indicating less than 25%, 2 indicating 26% to 50%, 3 indicating 50% to 75% and 4 indicating greater than over 75%. The staining intensity and the proportion of positive cells were combined through multiplication, leading to a final score ranging from 0 to 12. Staining with final scores between 0 and 6 was considered low expression, while final scores between 7 and 12 were considered high expression^29^. CD8^+^ T cell infiltration into tumors was evaluated by manually counting the number of positive cells in high-magnification fields (20×) for each sample the determine quantitative results. In addition, OTOP2 IHC images were downloaded from The Human Protein Atlas (HPA, https://www.proteinatlas.org/).

### 2.9. Transfection of siRNA and cell culture

The human COAD cell lines HCT116 ,SW620 and SW480 from were acquired from the cell bank of the Chinese Academy of Sciences in Beijing, China. The cells were confirmed with STR profiling and grown in Certified Fetal Bovine Serum (FBS) from VivaCell in Shanghai, China, following previous protocols. GeneChem (Shanghai, China) synthesized siRNAs (named Si-OTOP2-1 and Si-OTOP2-2), along with scrambled siRNA (referred to as Si-NC), as well as the vector and OTOP2 plasmids. Lipofectamine 2000 reagent (Invitrogen, Carlsbad, USA) was used to introduce siRNAs/Si-NCs and OTOP2 plasmids/vectors into HCT116 and SW680 cells following the manufacturer's instructions.

### 2.10. Quantitative real-time PCR (qRT‒PCR)

Total RNA was extracted from the cells with TRIzol (Invitrogen). The extracted RNA was reverse transcribed into cDNA using a PrimeScript RT reagent kit with gDNA Eraser from TaKaRa in Tokyo, Japan. qRT‒PCR was performed using TaKaRa's SYBR Premix Ex TaqII, with GAPDH utilized as an internal reference gene. Using the 2-ΔΔCt method, the relative gene expression levels were determined and are shown in Supplementary Table 3.

### 2.11. CCK-8 assays

A CCK-8 assay (Beyotime, China) was used to determine the proliferation of COAD cells. COAD cells were briefly seeded in 96-well plates at a density of 6×10^3^ cells per well. Following a 24-hour incubation period, 20 μl of CCK-8 cell proliferation reagent was added to each well, and the cells were then incubated for an additional 2 hours. The absorbance of the culture medium at 450 nm was measured using a microplate spectrophotometer from BioTek (VT, USA).

### 2.12. Transwell assay

We measured the migration or invasion of COAD cells with a Transwell assay (Corning, Inc.). COAD cells (3×10^4^) were transferred to the upper chamber using serum-free medium (Matrigel was included in the upper chambers for the invasion test before transferring the cells), while serum-free medium was added to the lower chamber. Following a 24-hour incubation at 37°C, the cells that did not migrate or invade the upper membrane were removed by washing. The cells that migrated or invaded were then treated with 4% paraformaldehyde (PFA) and stained with 0.1% crystal violet (Beyotime, China) 30. The total number of stained cells was counted.

### 2.13. Cell adhesion assays

Cell adhesion experiments were conducted with a Vybrant Cell Adhesion Assay Kit (Invitrogen, USA). Briefly, 100 µl of coating buffer was added to each well of a 96-well plate and then incubated overnight at 4°C. The next day, the buffer coating was removed, and the plate was rinsed three times with PBS^31^.

Next, 5 million cells per milliliter were seeded in a 96-well plate, with one well for each. The experimental wells consisted of four identical wells, while the control group included four wells that were not treated. The cells were incubated at 37°C for half an hour. Following incubation, the culture medium was retained in the control well while it was removed from the experimental well. Next, 100 µl of cell staining solution B was added to each well, followed by incubation of the cells at 37°C for 1 hour. The optical density (OD) at 450 nm was measured using a microplate reader. The percentage of adherent cells was calculated by the following formula: adherent cells (%) = (test well OD450-blank well OD450)/(control well OD450-blank well OD450)*100. A minimum of three repetitions were conducted for each experiment. The information is presented as the average proportion of cells that were adherent.

## 3. Results

### 3.1. Analysis of OTOP2 expression and survival in COAD patients

Using the TCGA database, we compared the mRNA expression of OTOP2 between tumor and normal tissue specimens from 33 kinds of tumors. As shown in Supplementary Figure 1A, *OTOP2* was significantly downregulated in 14 types of cancer, including glioblastoma multiforme (*P*<0.01, GBM), glioma (*P*<0.0001, GBMLGG), lower grade glioma (*P*<0.001, LGG), uterine corpus endometrial carcinoma (*P*<0.0001, UCEC), COAD (*P*<0.0001), COAD/rectal adenocarcinoma (*P<*0.0001,COAD/READ), prostate adenocarcinoma (*P<*0.05, PRAD), head and neck squamous cell carcinoma (*P*<0.01, HNSC), skin cutaneous melanoma (*P<*0.0001, SKCM), READ (*P*<0.0001), testicular germ cell tumors (*P*<0.0001, TGCT), uterine carcinosarcoma (*P*<0.0001, UCS), pheochromocytoma and paraganglioma (*P*<0.01, PCPG), and kidney chromophobe (*P*<0.0001, KICH). Significant alterations in OTOP2 mRNA expression were observed in the 10 total tumor samples and their corresponding normal samples from various cancers within the TCGA Pancancer database, including COAD (*P*<0.001), HNSC (*P*<0.05), KICH (*P*<0.01), kidney clear cell cancer (*P*<0.01, KIRC), kidney papillary cell cancer (KIRP, *P*<0.01), liver hepatocellular carcinoma (LIHC, *P*<0.05), lung squamous cell cancer (LUSC,* P*<0.001), READ (*P*<0.01), stomach adenocarcinoma (STAD,* P*<0.01), and UCEC (P<0.05), (Supplementary Figure 1B).

**Figure 2:**
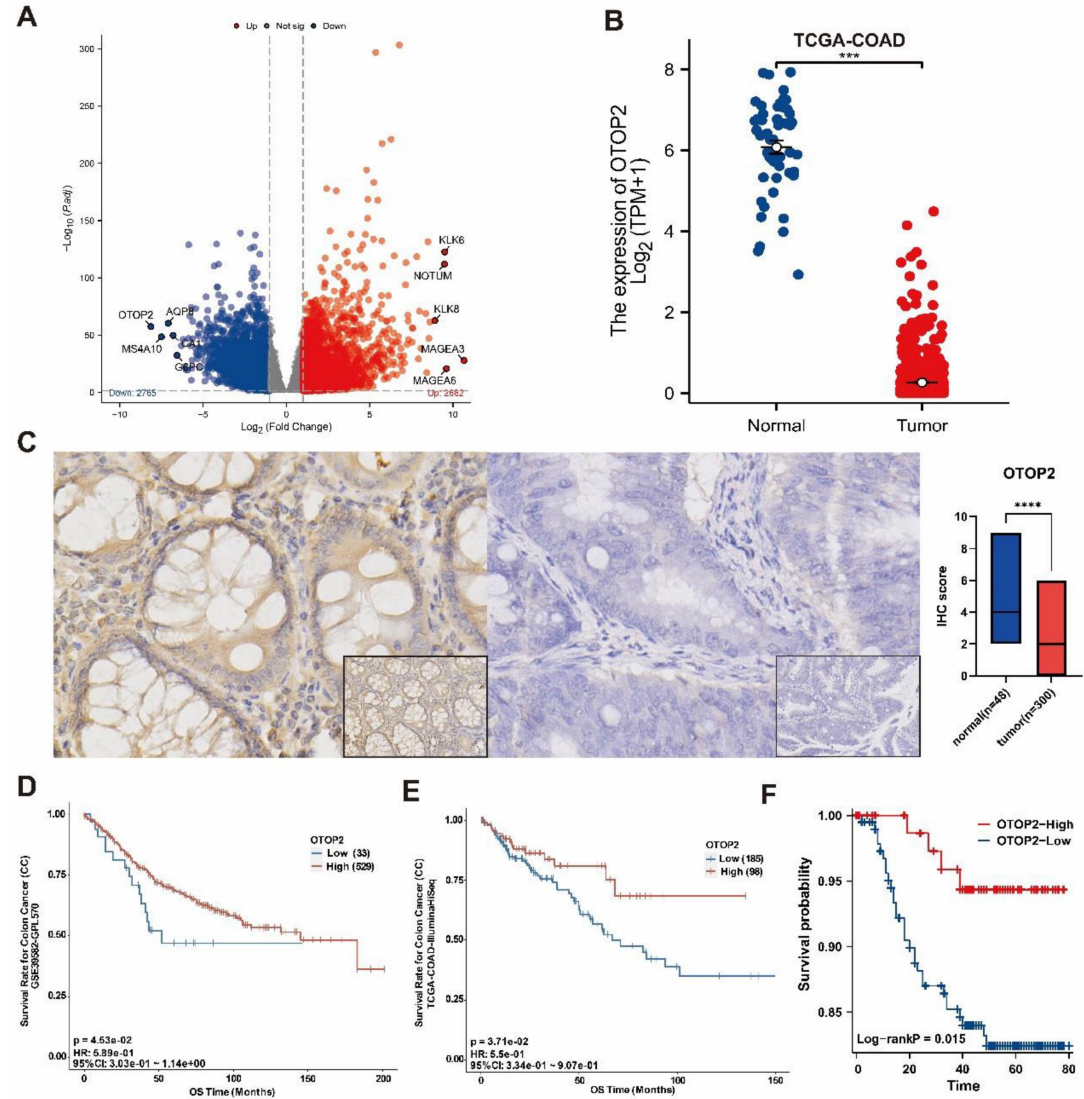
***OTOP2* Expression and survival analysis of in COAD. (A)** Volcano plot showed the obviously different mRNA expression between COAD and normal tissue from TCGA-COAD. The top five up-regulated or down-regulated genes, including *OTOP2*, were marked on the map. **(B)** The mRNA expression of* OTOP2* in COAD compared with normal tissues (n=444). **(C)** OTOP2 protein expression in cancerous and paired normal tissues from TMUCIH-COAD cohort. Magnification of main panel, 40×; Magnification of right bottom panel, 20×. **(D)** Overall survival curves of patients from GSE39582 according to the mRNA expression of *OTOP2*. **(E)** Overall survival curves of patients from TCGA-COAD according to the mRNA expression of *OTOP2*. **(F)** Overall survival curves of patients from TMUCIH cohort according to the protein expression of OTOP2. ***P < 0.001.

Additionally, our analysis revealed that OTOP2 was significantly downregulated in COAD tissues compared to normal tissues in the TCGA database, as shown in Figure 2A and 2B. Next, we confirmed the protein levels of OTOP2 in the TMUCIH-COAD group through IHC staining, and revealed a notable decrease in OTOP2 expression in COAD tissues compared to normal tissues in a cohort of 348 individuals (*P*<0.0001; Figure 2C). We obtained IHC images of OTOP2 from the HPA database, in which high expression of OTOP2 was dected in normal tissue and downregulation was detected in COAD (Supplementary Figure 2). Survival analysis revealed that patients with high OTOP2 mRNA expression had better OS than patients with low OTOP2 mRNA expression in the TCGA-COAD cohort (*P*=0.0453, Figure 2D) and in the GSE39582 cohort (*P*=0.0371; Figure 2E). A favorable prognostic role of high OTOP2 protein expression was confirmed for the OS of COAD patients in the TMUCIH-COAD cohort (*P*=0.047; Figure 2F). Our results demonstrated the significant downregulation of OTOP2 in COAD, indicating that OTOP2 downregulation might promote the development of COAD.

### 3.2. Abnormal OTOP2 mRNA expression was related to copy number alternations and promoter methylation

**Figure 3:**
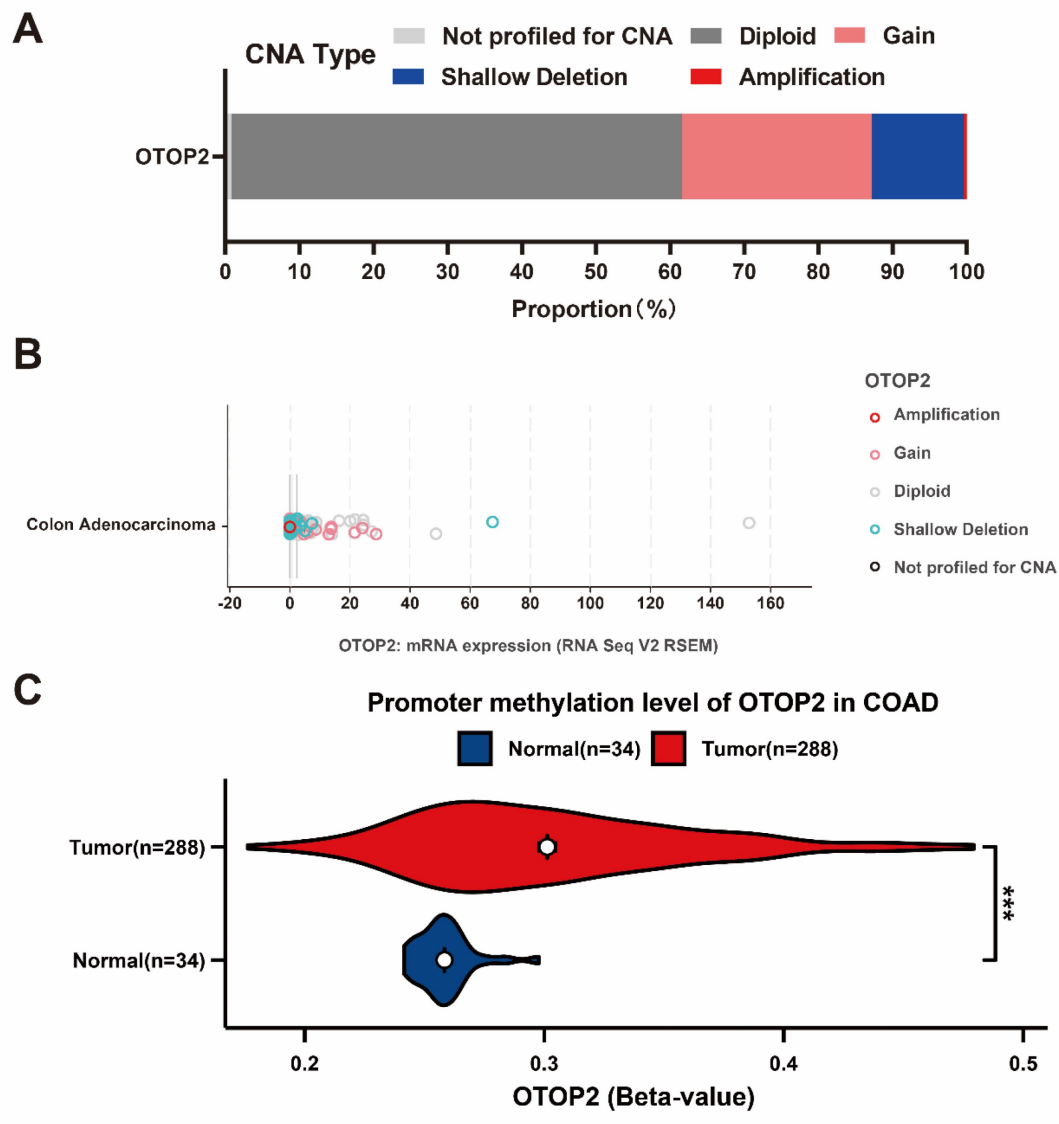
** Genetic alterations underly *OTOP2* dysregulation in COAD. (A)** The CNA proportion of *OTOP2* in TCGA-COAD. **(B)** The relationship between CNAs and mRNA expression of *OTOP2* in TCGA-COAD. **(C)** The promoter methylation levels of OTOP2 in COAD and normal tissue. ***P < 0.001.

To clarify the cause of OTOP2 dysregulation, we performed a thorough examination of CNA and DNA methylation. As shown in Figure 3A, in the cBioPortal database, OTOP2 shallow deletions were detected in approximately 12.4% of COAD patients, gain in 25.62%, and amplification in 0.41%. Moreover, combined with the TCGA-COAD RNA-seq data, we found that shallow deletions were mainly enriched in patients with low OTOP2 expression (Figure 3B). Considering that abnormal DNA methylation is a hallmark of cancer cells and can impact protein expression^32^, we compared OTOP2 promoter methylation levels between COAD tissue and normal tissue using the UACLAN tool. The results demonstrated that OTOP2 promoter methylation was significantly increased in COAD tissue (*P*<0.001; Figure 3C). Therefore, OTOP2 downregulation might result from a multidimensional process that involves OTOP2 CNA and promoter methylation in COAD.

### 3.3. OTOP2 inhibited the proliferation, migration, invasion and adhesion of COAD cells

**Figure 4:**
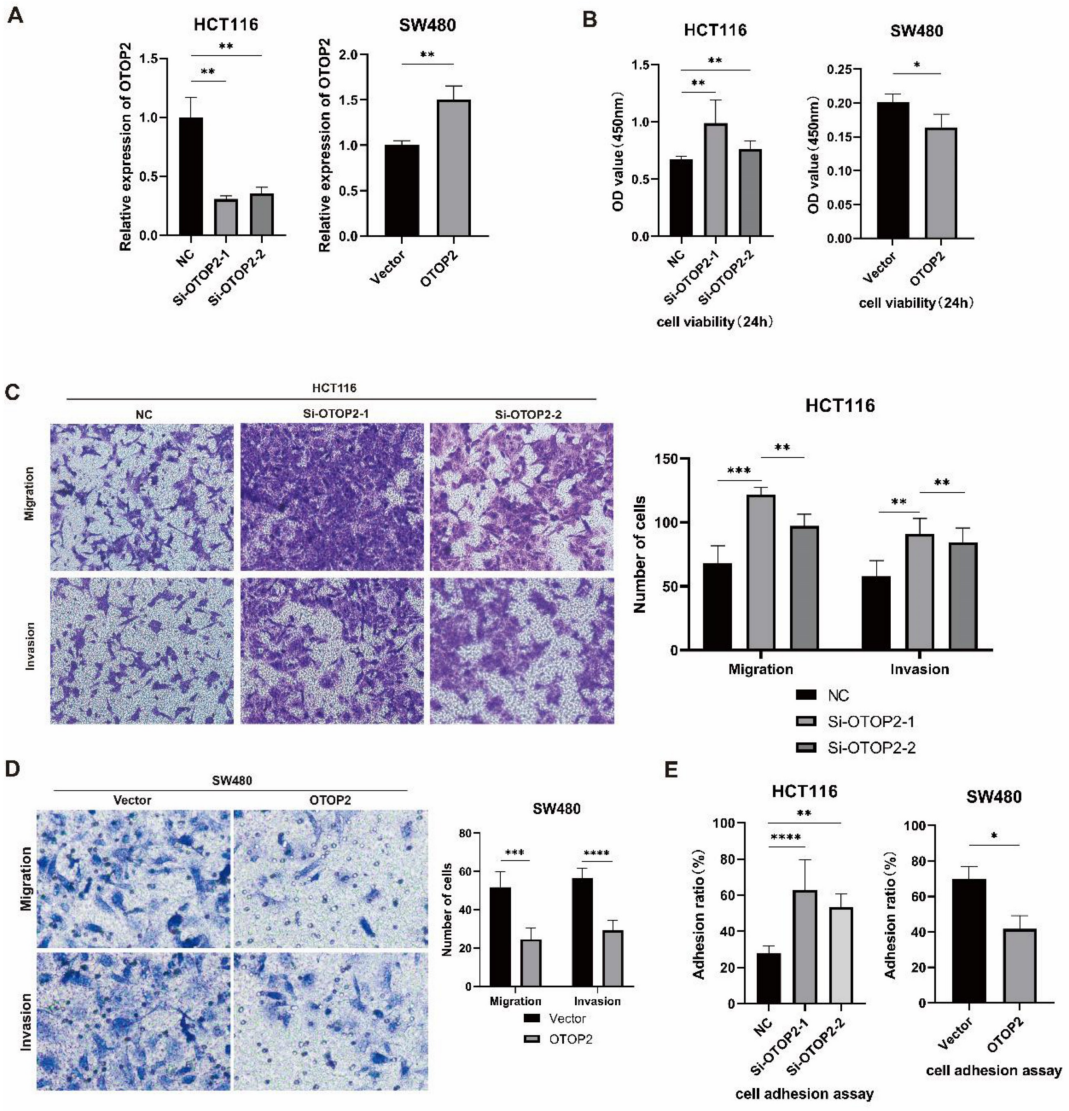
** The expression level of *OTOP2* affected the proliferation, migration, invasion and adhesion of COAD cells.** HCT116 cells were transfected with si-NC, si-*OTOP2*-1, and si-*OTOP2*-2 plasmids, and SW480 cells were transfected with vector or *OTOP2* plasmids. The mRNA expression level of *OTOP2* was evaluated by qRT‒PCR 24 hours after transfection. **(B)** The proliferation of HCT116 and SW480 cells was determined using a CCK-8 assay. **(C)** Transwell migration and invasion assays were performed to evaluate the migration and invasion ability of HCT116 cells. **(D)** Transwell migration and invasion assays were performed to evaluate the migration and invasion ability of SW480 cells. **(E)** The adhesion assay kit was used to determine the adhesion ability of HCT116 and SW480 cells. The presented data were obtained from at least three independent experiments and are expressed as the mean ± SD. Statistical analysis was performed using one-way ANOVA and 2-tailed Student's t test to determine statistical significance. *P < 0.05, **P < 0.01, ***P < 0.001, ****P < 0.0001.

Because OTOP2 expression was downregulated in COAD tissues, we investigated whether OTOP2 knockdown affects the cellular functions of COAD cells *in vitro*. We first examined endogenous OTOP2 expression in HCT116, SW480 and SW620 cell lines and found that its expression was the highest in HCT116 cells and the lowest in SW480 cells (Supplementary Figure 3). Thus, we knocked down OTOP2 in HCT116 cells using siRNA and overexpressed OTOP2 in SW480 cells using an OTOP2 plasmid (Figure 4A).

We performed a CCK-8 proliferation assay and observed greater cell proliferation in the Si-OTOP2-1 and Si-OTOP2-2 HCT116 cells than in the control cells (Si-NCs), whereas the overexpression of OTOP2 in SW480 cells significantly inhibited cell proliferation (Figure 4B). Additionally, there was an increase in migration and invasion in both Si-OTOP2-1 and Si-OTOP2-2 HCT116 cells, whereas the overexpression of OTOP2 inhibited the migration and invasion of SW480 cells (Figure 4C, D). Adhesion was greater in the Si-OTOP2-1 and Si-OTOP2-2 groups than in the Si-NC group in adhesion assays, but adhesion was notably reduced in SW480 cells after OTOP2 overexpression (Figure 4E). In COAD, *in vitro* studies showed that OTOP2 suppressed the growth, movement, penetration, and attachment of tumor cells.

### 3.4. High levels of OTOP2 were linked to increased immune pathway activity and infiltration of immune cells in patients with COAD

**Figure 5:**
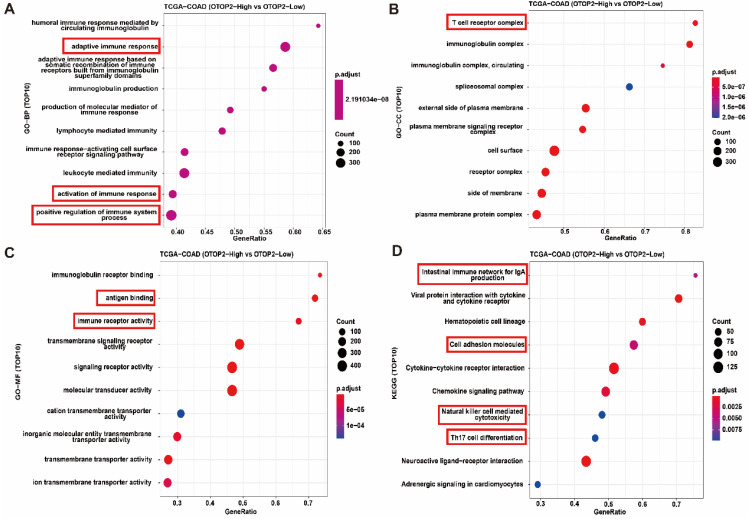
** Functional enrichment indicated that *OTOP2* expression was associated with immune related pathways. (A)** Enrichment analysis of TOP10 GO-BP terms of *OTOP2* related genes in TCGA-COAD. **(B)** Enrichment analysis of TOP10 GO-CC terms of *OTOP2* related genes in TCGA-COAD. **(C)** Enrichment analysis of TOP10 GO-MF terms of *OTOP2* related genes in TCGA-COAD. **(D)** Enrichment analysis of TOP10 KEGG terms of *OTOP2* related genes in TCGA-COAD.

To determine the molecular mechanism of OTOP2 in the tumorigenesis and progression of COAD, we conducted enrichment analyses by using CAMOIP. First, the GSEA results indicated that OTOP2 was involved in several pathways, including the cell cycle checkpoint, G2/M checkpoint, and mitotic spindle checkpoint pathways (Supplementary Table 4). GO-BP enrichment analyses revealed that genes associated with OTOP2 were primarily enriched in the adaptive immune response pathway, immune response, and positive regulation of immune system processes (Figure 5A). GO-CC enrichment analysis revealed that genes related to OTOP2 were mainly enriched in the T cell receptor complex and immunoglobulin complex, as shown in Figure 5B. GO-MF enrichment analysis revealed that genes related to OTOP2 were enriched in pathways related to antigen binding and immune receptor activity (Figure 5C). KEGG enrichment analyses indicated that genes related to OTOP2 were predominantly enriched in the intestinal immune network associated with IgA production, cell adhesion molecules, natural killer cell-mediated cytotoxicity, and Th17 cell differentiation (Figure 5D). Therefore, these results indicate that OTOP2 is involved in immune regulation.

**Figure 6:**
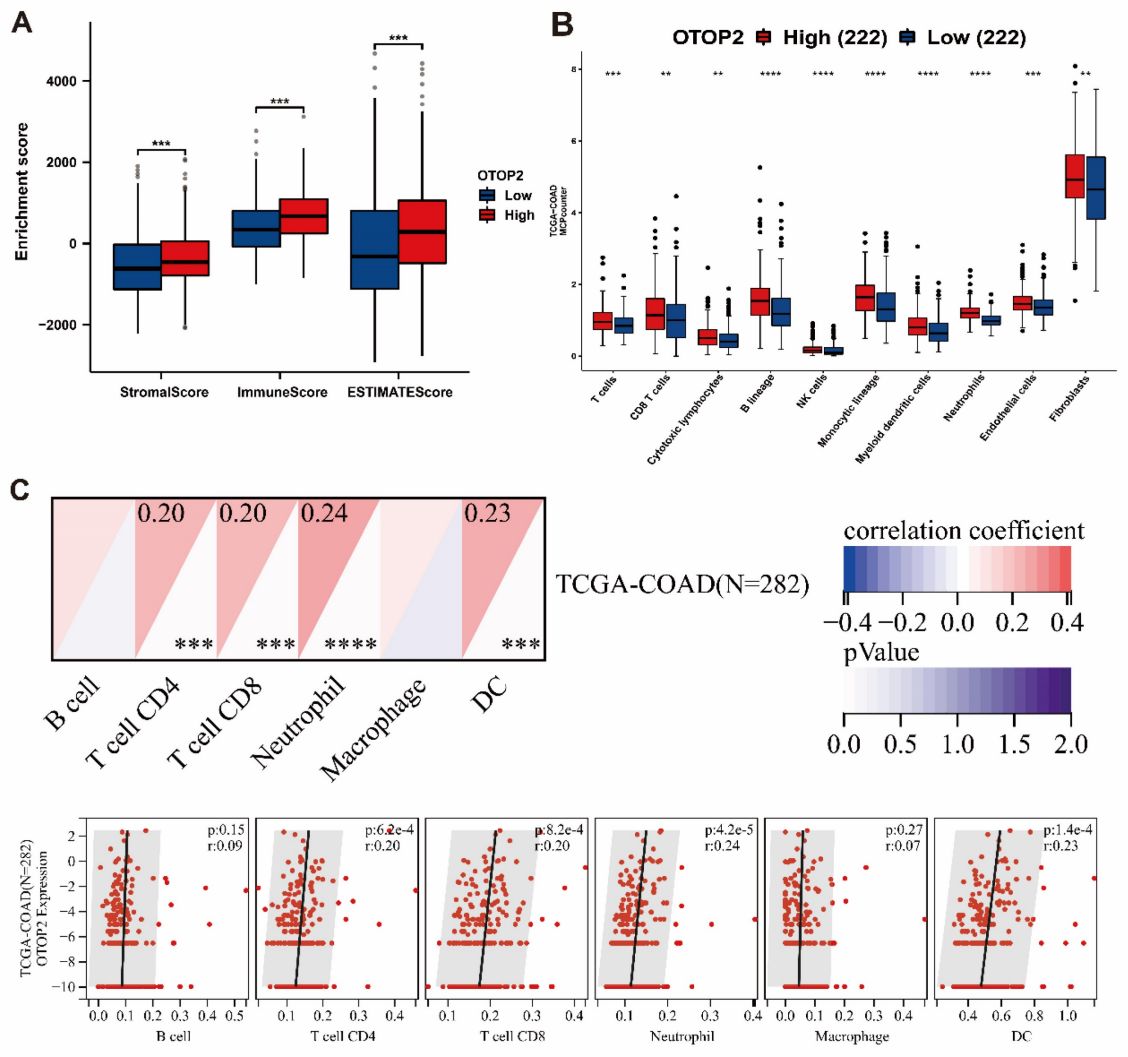
** Immune infiltration algorithm revealed the correlations of *OTOP2* expression with immune cells in COAD. (A)** Comparison of stromal Score, immune Score and ESTIMATE score between high and low *OTOP2* mRNA expression in TCGA-COAD (n=444). **(B)** Comparison of immune infiltration level between high and low *OTOP2* mRNA expression from CAMOIP (n=444). **(C)** Heatmap and scatter plot representing the correlation between immune cells infiltration and *OTOP2* mRNA expression from SangerBox (n=282). ns *P* ≥ 0.05, *P < 0.05, **P < 0.01, ***P < 0.001, ****P < 0.0001.

The presence of immune cell infiltration in the TME is linked to tumorigenesis, progression or metastasis^33^,^34^. Our examination of patients with COAD, revealed that the expression of OTOP2 was linked to increased immune infiltration based on the stromal score (*P*<0.001), immune score (*P*<0.001), and ESIMATE score (*P*<0.001) (Figure 6A). Next, we used the MCPcounter algorithm to examine the presence of immune cell populations in the OTOP2 groups with high and low expression levels. The findings indicated a notable increase in immune cell infiltration in the high OTOP2 expression group compared to the low OTOP2 expression group, as demonstrated in Figure 6B. By utilizing SangerBox web tools, we showed that there was a notable association between the OTOP2 expression level and the presence of immune cells (Figure 6C), such as CD4^+^ T cell (*R*=0.20, *P*=6.2e-4), CD8^+^ T cell (*R*=0.20, *P*=8.2e-4), neutrophils (*R*=0.24, *P*=4.2e-5), and dendritic cells (DCs) (*R*=0.23, *P*=1.4e-4). The analysis of the enrichment and infiltration of immune cells mentioned earlier suggested that OTOP2 could impact the progression and advancement of COAD by influencing the immune environment within tumors.

### 3.5. OTOP2 expression was correlated with antigen presentation pathway activity and CD8^+^ T cell infiltration in COAD

**Figure 7:**
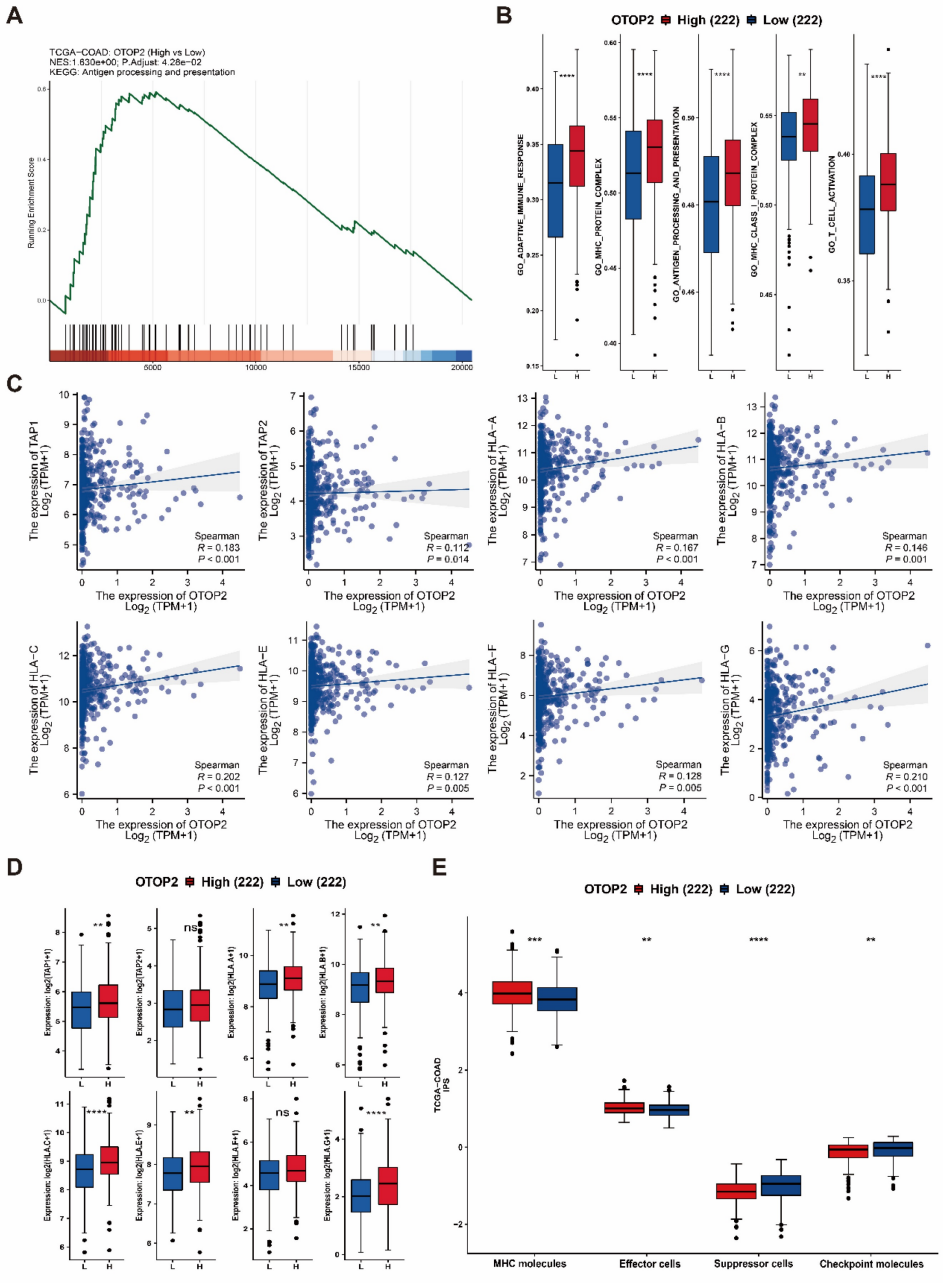
** Relationships between *OTOP2* mRNA expression and antigen presentation pathways as well as immune checkpoints. (A)** GSEA showed that *OTOP2* related genes from TCGA-COAD involved in antigen presentation pathway. **(B)** ssGSEA algorithm showed the different scores of adaptive immune response, MHC protein complex, antigen processing and presentation, MHC Class I protein complex and T cell activation between high* OTOP2* expression (H) and low OTOP2 expression (L) samples in TCGA-COAD. **(C)** Timer 2.0 webtool showed that *TAP1, TAP2* and *HLA-A/B/C/D/E/F/G MHC-I* (encoding MHC I protein), were positively correlated with *OTOP2* mRNA expression in TCGA-COAD samples (n=444). **(D)** Differences in antigen presentation-related molecules (TAP1, TAP2 and HLA-A/B/C/D/E/F/G) between high and low *OTOP2* expression samples in TCGA-COAD. L Low, H High. **(E)** Comparison of the mRNA expression of MHC molecules, effector cells, suppressor cells and checkpoint molecules between high and low *OTOP2* expression samples in TCGA-COAD. ns *P* ≥ 0.05, *P < 0.05, **P < 0.01, ***P < 0.001, ****P < 0.0001.

GSEA suggested that pathways related to antigen processing and presentation were enriched in the TCGA-COAD cohort with high expression of OTOP2 (*P*=0.0428; Figure 7A). Furthermore, individuals with elevated OTOP2 levels exhibited enhanced adaptive immune reactions, MHC protein complex expression, antigen processing and presentation, MHC class I protein complex expression, and T cell activation (Figure 7B). These pathways involve key immune molecules such as MHC-I, TAP1, and TAP2^35^. Further examination revealed a direct relationship between the levels of OTOP2 and the genes responsible for TAP1, TAP2, and MHC-I (Figure 7C, D), suggesting a possible link between OTOP2 expression and the immune response to tumors. A pancancer heatmap analysis further confirmed the positive correlation between OTOP2 expression and the expression of MHC molecules (Supplementary Figure 4). The IPS indicated a direct relationship between OTOP2 gene expression and MHC molecule and effector cell expression, but an inverse relationship between OTOP2 gene expression and suppressor cell and checkpoint molecule expression was also noted.

**Figure 8:**
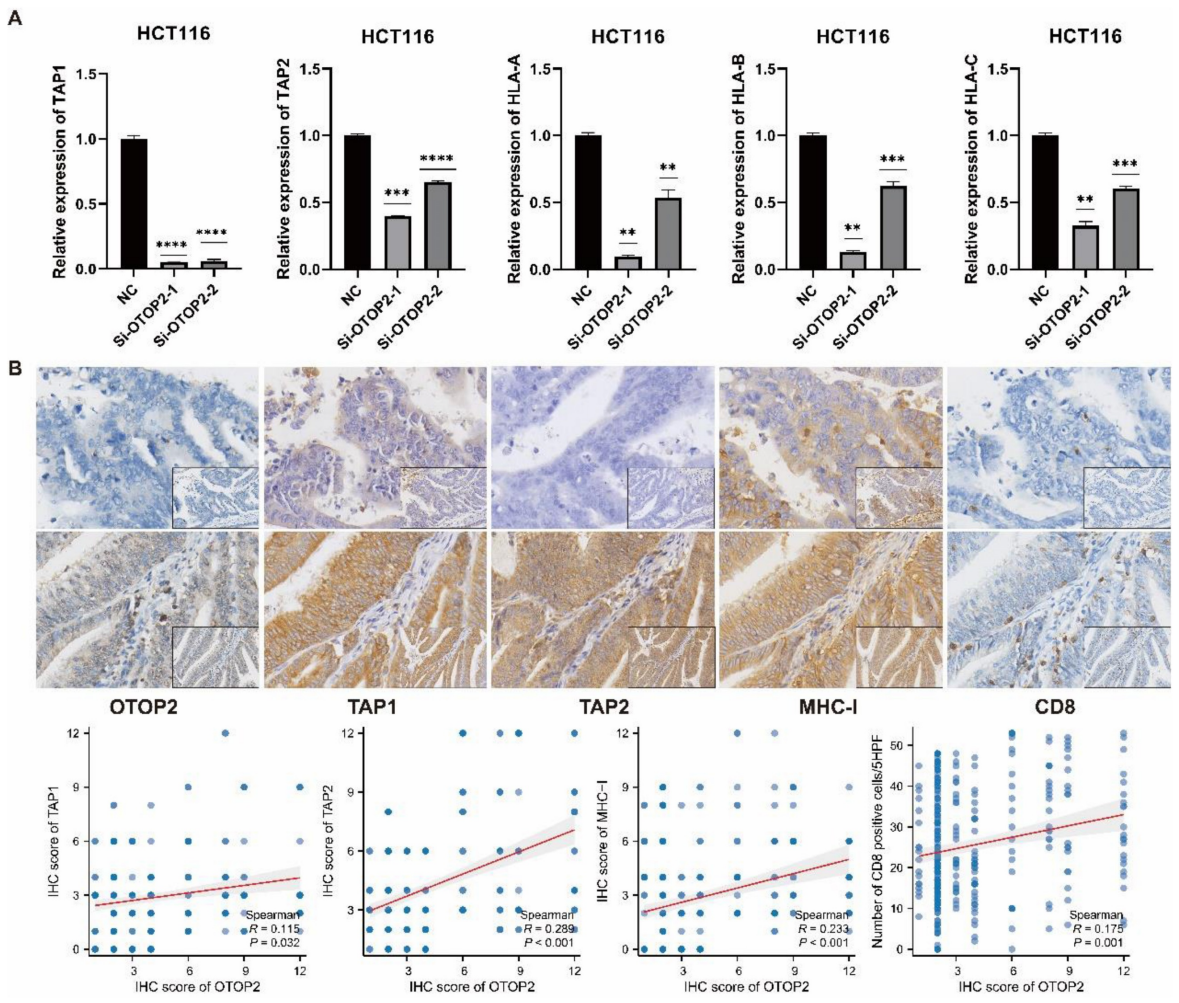
** The validation the effect of OTOP2 expression on TAP1, TAP2 and MHC-I expression and CD8 infiltration in COAD. (A)** HCT116 cells were transfected with plasmids Si-NC or Si-OTOP2-1 and Si-OTOP2-2, and the expression levels of TAP1, TAP2 and HLA-A/B/C were determined by qRT-PCR at 24 h post-transfection. **(B)** Expression of OTOP2, TAP1, TAP2, MHC-I and CD8 and their correlations in 348 cases of TMUCIH-COAD cohort (n=348). Magnification of main panel, 40×; Magnification of right bottom panel, 20×. The data presented are representative of at least three independent experiments and are expressed as mean ± SD. Statistical significance was determined using a one-way ANOVA and 2-tailed Student's t test. **P < 0.01, ***P < 0.001, ****P < 0.0001.

We then performed *in vitro* experiments to validate the effect of OTOP2 mRNA expression on MHC molecules in the COAD cell line. The knockdown of OTOP2 in HCT116 cells significantly reduced the expression of TAP1, TAP2, and HLA-A/B/C (Figure 8A). The IHC results also demonstrated that low expression of OTOP2 was associated with decreased expression of TAP1 (*R*=0.115, *P*=0.032), TAP2 (*R*=0.289, *P*<0.001), and MHC-I (*R*=0.233, *P*<0.001), as well as decreased CD8^+^ T cell infiltration (*R*=0.175, *P*=0.001) in COAD tissue (Figure 8B). Bioinformatics analysis, *in vitro* experiments and IHC analysis suggested that OTOP2 expression modulates MHC expression and subsequent CD8^+^ T cell infiltration in COAD.

## 4. Discussion

COAD has the highest morbidity and related deaths among gastrointestinal tumors worldwide^36^,^37^. The pathogenesis of COAD is relatively well-known and involves the accumulation of multiple genetic abnormalities^38^. Analysis of important molecular abnormalities can help further elucidate the mechanism of CRC development and progression and provide potential therapeutic targets.

Recently, OTOP2 was found to be a conserved sequence-selective ion channel protein found mainly in the vestibular, gustatory, and digestive systems of vertebrates. It could be involved in detecting pH changes within cells for signaling or regulating development^39^-^41^. However, its functions and related mechanisms in tumors have rarely been reported until recently, when relatively low expression of OTOP2 was observed in ulcerative colitis and throughout the progression to COAD compared with that in normal intestinal epithelium^11^,^12^. In this study, through bioinformatics analyses of many samples from the TCGA database and case verification using the TMUCIH-COAD cohort, we demonstrated the downregulation of OTOP2 in COAD. Moreover, our study showed that COAD patients with high OTOP2 expression had more favorable overall survival than those with low OTOP2 expression, consistent with the conclusions of previous studies 10 12. Furthermore, our investigation revealed that OTOP2 downregulation might result from a multidimensional process that involves shallow OTOP2 deletion and promoter methylation in COAD.

Guo *et al.* 12 reported that OTOP2 suppressed the growth, spread, and movement of colorectal cancer cells, suggesting that OTOP2 acts as a tumor suppressor targeted by miR-3148. This study not only demonstrated that OTOP2 could inhibit cell proliferation, invasion, and migration, but also reduced cancer cell adhesion. Adhesion between cancer cells is crucial for the aggressive behavior of tumor cells and the colonization of distant metastatic lesions^44^-^46^. Most importantly, for the first time, we revealed a close association between OTOP2 expression and the tumor immune micro-environment. In recent years, immunotherapy has demonstrated potential for patients with COAD; however, only a minority of patients with MSI-H or dMMR tumors see exhibit positive results from immune checkpoint inhibitors^47^. In addition to affecting tumor cells, the tumor immune microenvironment could have a significant impact on the immune response and evasion, ultimately influencing tumor development and treatment results. The research revealed a positive association between OTOP2 expression levels and immune effector cells (such as T cell, DC, and neutrophil), as well as a negative correlation with immune-suppressive factors (such as immune checkpoints) based on the IPS. Additionally, our bioinformatics studies revealed that OTOP2 plays a role in controlling the antigen presentation process, a finding that has not been previously reported. The MHC-I complex plays a vital role in the immune system's ability to recognize and destroy cancer cell through CD8^+^ T cell in the antigen presentation pathway^48^.

In general, CD8^+^ T cell are believed to promote the effectiveness of immune checkpoint therapy. Ribas *et al.* suggested that resistance to immune checkpoint therapy mainly stems from changes in the antigen presentation pathway in tumor cells which also impaires the antitumour activity of CD8^+^ T cell^49^. With the help of TAP1 and TAP2, CD8^+^ T cell can identify MHC-I molecules on the exterior of tumor cells, resulting in the destruction of tumor cells^50^. Tumeh *et al.* demonstrated a correlation between the number of CD8^+^ T cell and tumor regression in 46 COAD patients who received PD-1 blockade therapy and the density of CD8^+^ T cell was greater at the tumor margins^51^. These mechanisms aid in preventing tumor invasion and dissemination^52^,^53^. Cancer cells often use the strategy of reducing MHC-I expression to evade immunotherapy^54^-^58^.

Subsequent *in vitro* experiments and TMA validation confirmed the effect of OTOP2 expression on TAP1, TAP2 and MHC-I expression as well as CD8^+^ T cell infiltration. Based on these results, we hypothesized that OTOP2 in COAD cells might modulate the expression and function of MHC-I and further affect the recognition and killing effects of CD8^+^ T cell on tumor cells. Additionally, patients with COAD who have elevated OTOP2 levels may experience improvements in response to immunotherapy. However, this hypothesis requires validation through in-depth *in vivo* experiments and clinical trials.

## 5. Conclusion

Our research showed that OTOP2 functions as a tumor suppressor in COAD, suggesting its potential as a prognostic biomarker for patients with this condition. Additionally, our initial findings revealed a strong association between OTOP2 expression and the MHC-I antigen presentation pathway as well as immune cell infiltration, indicating that OTOP2 expression could serve as a reliable indicator of immunotherapy effectiveness.

## Supplementary Material

Supplementary figures and tables.

## Abbreviations

OTOP2: Otopetrin 2
COAD: colon adenocarcinoma
TCGA: The Cancer Genome Atlas
TMAs: tissue microarrays
TME: tumor microenvironment
IPS: immunophenoscore
MHC: major histocompatibility complex
IHC: immunohistochemical
OS: overall survival
GEO: Gene Expression Omnibus
CNA: copy number alterations
DEGs: differently expressed genes
GO: Gene Ontology
KEGG: Kyoto Encyclopedia of Genes and Genomes
GSEA: Gene Set Enrichment Analysis
FDR: false discovery rate
ssGSEA: Single-sample GSEA
CAMOIP: Comprehensive Analysis on Multi-Omics of Immunotherapy in Pancancer
FBS: Fetal Bovine Serum
qRT‒PCR: Quantitative real-time PCR
PFA: paraformaldehyde
OD: optical density
GBM: glioblastoma multiforme
GBMLGG: glioma
LGG: lower grade glioma
UCEC: uterine corpus endometrial carcinoma
READ: rectal adenocarcinoma
PRAD: prostate adenocarcinoma
HNSC: head and neck squamous cell carcinoma
SKCM: skin cutaneous melanoma
TGCT: testicular germ cell tumors
UCS: uterine carcinosarcoma
PCPG: pheochromocytoma and paraganglioma
KICH: kidney chromophobe
ESCA: esophageal cancer
KIRP: kidney papillary cell cancer
KIPAN: Pan-kidney cohort
STAD: stomach adenocarcinoma
KIRC: kidney clear cell cancer
LUSC: lung squamous cell cancer
WT: Wilms' tumor
OV: ovarian serous cystadenocarcinoma
ALL: acute lymphoblastic leukemia
LAML: acute myeloid leukemia
ACC: adrenocortical cancer
DCs: dendritic cells
