# Supplementary material for: Determination of the Immunomodulatory Role of OTOP2 in Colon Adenocarcinoma
Source: J Cancer. 2024 Jul 16;15(15):4838–52. doi: 10.7150/jca.95622 (PMC11310881; doi:10.7150/jca.95622)
Supplement: Supplementary file 1 — Supplementary figures and tables. [file jcav15p4838s1.pdf]

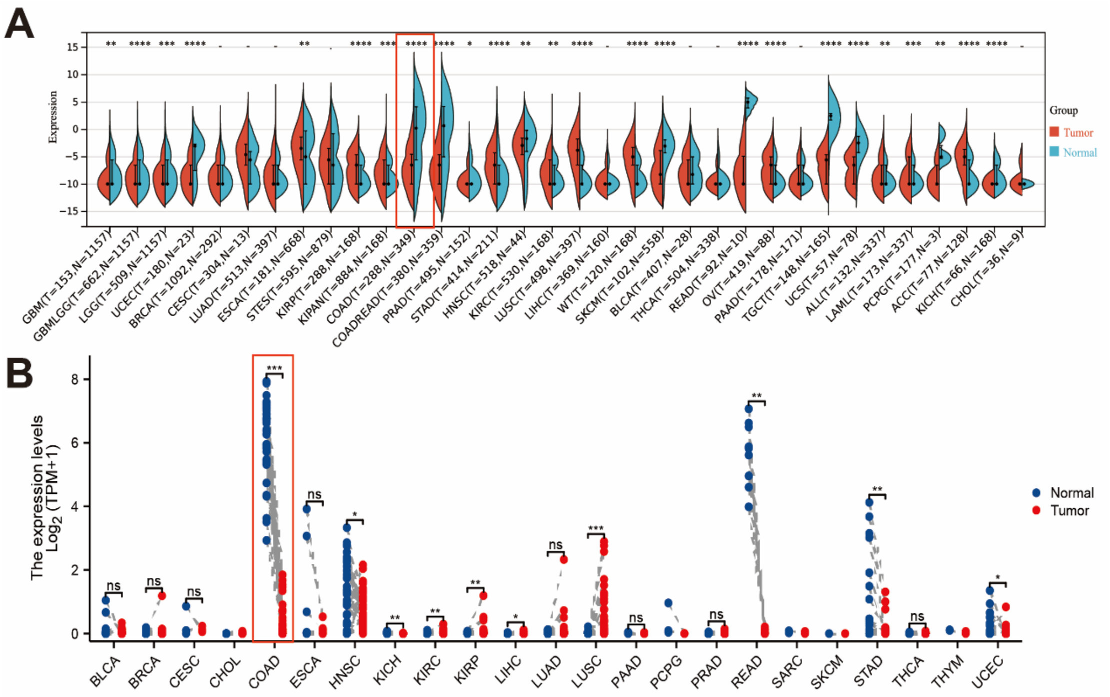

Supplementary Figure 1

Aberrant mRNA expression of *OTOP2* in TCGA pan-cancer.

(A) Comparison of OTOP2 mRNA expression between tumor and normal specimens in 33 kinds of cancers. (B) Comparison of OTOP2 mRNA expression between tumor and paired normal specimens in 23 kinds of cancers ns, $P \geq 0.05$, *$P < 0.05$, **$P < 0.01$, ***$P < 0.001$, ****$P < 0.0001$.

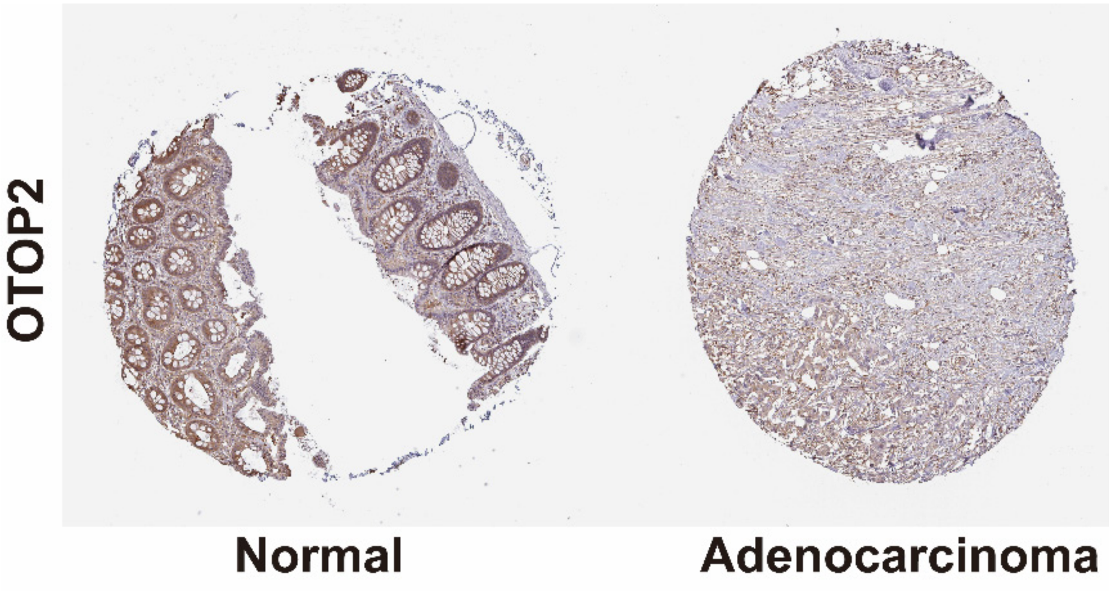

Supplementary Figure 2

Immunohistochemistry staining images of OTOP2 in normal colon mucosa and COAD from HPA dataset.

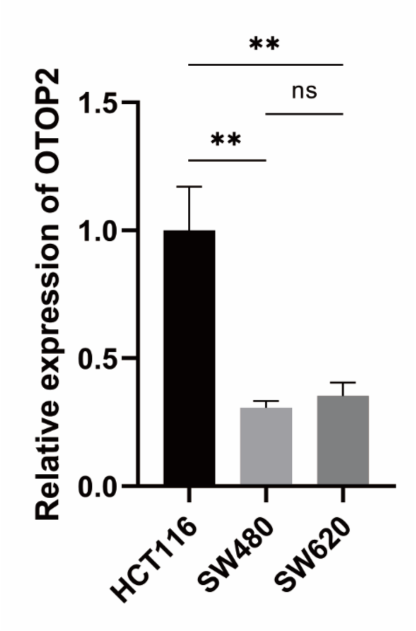

Supplementary Figure 3

***OTOP2* endotogenetic expression in HCT116, SW480 and SW620 COAD cell lines.**

The relative expression of *OTOP2* in different COAD cell lines was detected using qRT-PCR. ns, $P \geq 0.05$, $^{**}P < 0.01$.

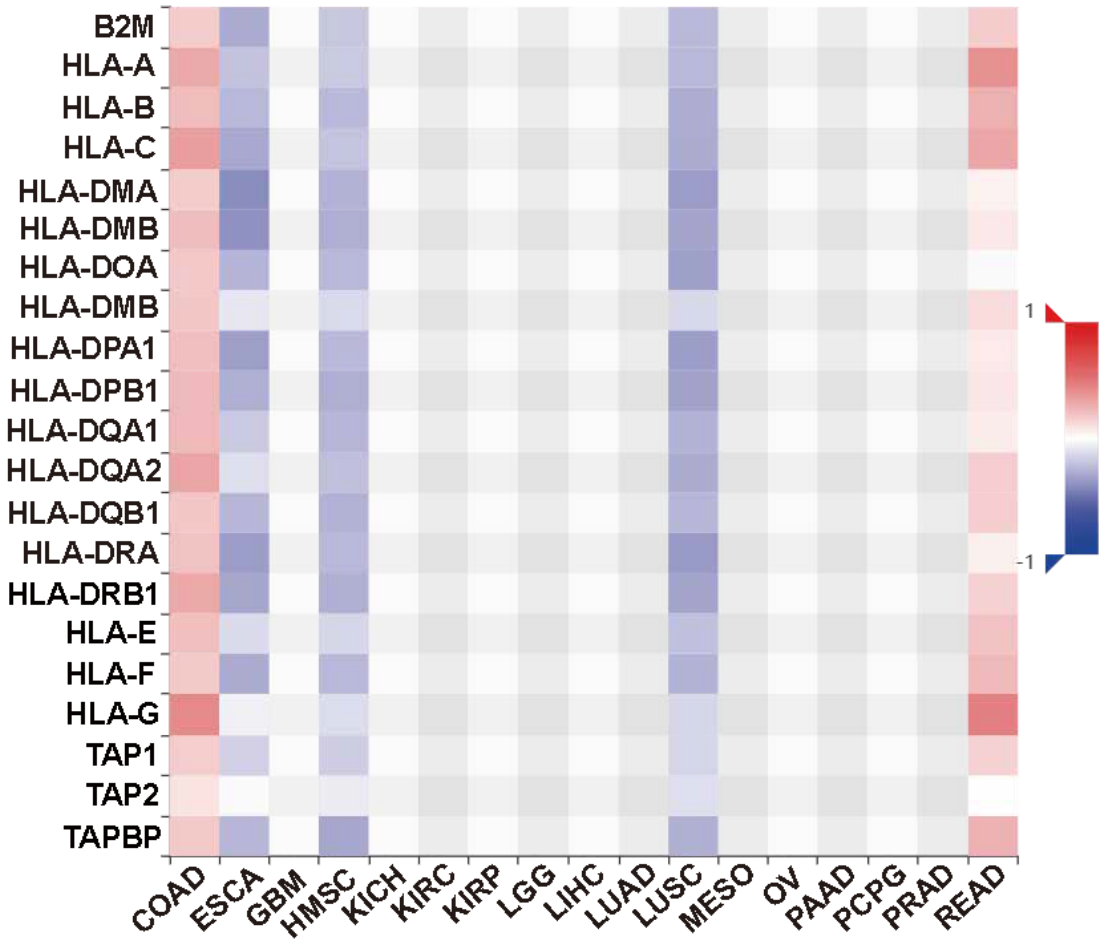

Supplementary Figure 4

Correlation of *OTOP2* expression with the expression of MHC molecules in pan-cancer from TISIDB.

Supplementary Table 1 Clinical characteristics of the TCGA-COAD patients

characteristics	Low expression of OTOP2	High expression of OTOP2
n	226	228
Pathologic T stage, n (%)		
T1	6 (1.3%)	5 (1.1%)
T2	42 (9.3%)	35 (7.7%)
T3	148 (32.7%)	161 (35.5%)
T4	29 (6.4%)	27 (6%)
Pathologic N stage, n (%)		
N0	129 (28.4%)	138 (30.4%)
N1&N2	97 (21.4%)	90 (19.8%)
Pathologic M stage, n (%)		
M0	158 (39.8%)	175 (44.1%)
M1	29 (7.3%)	35 (8.8%)
Gender, n (%)		
Male	126 (27.8%)	114 (25.1%)
Female	100 (22%)	114 (25.1%)
Race, n (%)		
Asian	9 (3.2%)	2 (0.7%)
Black or African American	34 (12.1%)	25 (8.9%)
White	127 (45%)	85 (30.1%)
Histological type, n (%)		
Mucinous adenocarcinoma	30 (6.7%)	32 (7.1%)
Adenocarcinoma	192 (42.8%)	195 (43.4%)
Age, n (%)		
<= 65	96 (21.1%)	92 (20.3%)
> 65	130 (28.6%)	136 (30%)

Supplementary Table 2 Clinical characteristics of the TMUCIH-COAD patients

characteristics	Low expression of OTOP2	High expression of OTOP2
n	265	83
Pathologic T stage, n (%)		
T1	3 (0.9%)	0 (0%)
T2	36 (10.3%)	9 (2.6%)
T3	164 (47.1%)	52 (14.9%)
T4	62 (17.8%)	22 (6.3%)
Pathologic N stage, n (%)		
N0	167 (48%)	58 (16.7%)
N1&N2	98 (28.2%)	25 (7.2%)
Pathologic M stage, n (%)		
M0	247 (71%)	75 (21.6%)
M1	18 (5.2%)	8 (2.3%)
Gender, n (%)		
male	127 (36.5%)	48 (13.8%)
female	138 (39.7%)	35 (10.1%)
Race, n (%)		
Asian	265(76.2%)	83(23.9)
Histological type, n (%)		
Mucinous Adenocarcinoma	107 (30.7%)	34 (9.8%)
Adenocarcinoma	158 (45.4%)	49 (14.1%)
Age, n (%)		
<= 65	189 (54.3%)	68 (19.5%)
＞65	76 (21.8%)	15 (4.3%)

Supplementary Table 3 Primers used for qRT-PCR analysis

Gene name	Primers (5′ –3′)
OTOP2	Forward-ACATCGAGTACAGCCTCTTCG
	Reverse-CGCTCACTTGAACCTCGTAGAT
TAP1	Forward-AAAGACACTCAACCAGAAGGAG
	Reverse-CACCAATGTAGAGGATTCCCAC
TAP2	Forward-TGGACGCGGCTTTACTGTG
	Reverse-GCAGCCCTCTTAGCTTTAGCA
HLA-A	Forward-GACGCCCCCAAAACGCATA
	Reverse-TGGGCAAACCCTCATGCTG
HLA-B	Forward-CAGTTCGTGAGGTTCGACAG
	Reverse-CAGCCGTACATGCTCTGGA
HLA-C	Forward-CCATGAGGTATTTGTGGACCG
	Reverse-TCTCGGACTCTCGTCGTCG
GAPDH	Forward-GGAGCGAGATCCCTCCAAAAT
	Reverse-GGCTGTTGTCATACTTCTCATGG

Supplementary Table 4 The GSEA enrichment results related to *OTOP2*

ID	Description	Enrichment Score	*P*.adjust
GO:0000075	cell cycle checkpoint	-0.36974	0.035899
R-HSA-68877	Mitotic Prometaphase	-0.43368	0.000909
R-HSA-68882	Mitotic Anaphase	-0.35777	0.040929
R-HSA-69473	G2/M DNA damage checkpoint	-0.5478	0.019441
R-HSA-69481	G2/M Checkpoints	-0.41192	0.04001
R-HSA-69618	Mitotic Spindle Checkpoint	-0.46972	0.007013
R-HSA-69620	Cell Cycle Checkpoints	-0.38174	0.003471
R-HSA-2555396	Mitotic Metaphase and Anaphase	-0.35893	0.035459
